# Supplementary material for: A C/EBPα–Wnt connection in gut homeostasis and carcinogenesis
Source: Life Sci Alliance. 2018 Dec 26;2(1):e201800173. doi: 10.26508/lsa.201800173 (PMC6306571; doi:10.26508/lsa.201800173)
Supplement: Supplementary file 1 [file LSA-2018-00173_TableS1.docx]

|  | % CEBPa positive area | nuclear C/EBPa | expression scoring | sample number |
| --- | --- | --- | --- | --- |
| adenocarcinoma | 60 | weak | 1 | AdCA case #1 |
|  | 60 | weak | 1 | AdCA case #2 |
|  | 80 | weak | 1 | AdCA case #3 |
|  | 90 | strong | 3 | AdCA case #4 |
|  | 100 | moderate | 2 | AdCA case #5 |
|  | 20 | weak | 1 | AdCA case #6 |
|  | 80 | weak | 1 | AdCA case #7 |
|  | 90 | moderate | 2 | AdCA case #8 |
|  | 100 | moderate | 2 | AdCA case #9 |
|  | 5 | weak | 1 | AdCA case #10 |
|  | 80 | moderate | 2 | AdCA case #11 |
|  |  |  |  |  |
| adenoma | 80 | moderate | 2 | Ad case #1 |
|  | 60 | moderate | 2 | Ad case #2 |
|  | 80 | moderate | 2 | Ad case #3 |
|  | 60 | weak | 1 | Ad case #4 |
|  | 80 | strong | 3 | Ad case #5 |
|  | 60 | moderate | 2 | Ad case #6 |
|  | 80 | moderate | 2 | Ad case #7 |
|  | 80 | moderate | 2 | Ad case #8 |
|  |  |  |  |  |
| Healthy/normal | 100 | weak | 1 | H&N case #1 |
|  | 100 | moderate | 2 | H&N case #2 |
|  | 100 | moderate | 2 | H&N case #3 |
|  | 100 | moderate | 2 | H&N case #4 |
|  | 80 | weak | 1 | H&N case #5 |
|  | 90 | weak | 1 | H&N case #6 |
|  | 100 | moderate | 2 | H&N case #7 |
|  | 100 | moderate | 2 | H&N case #8 |
|  | 100 | moderate | 2 | H&N case #9 |
|  | 100 | moderate | 2 | H&N case #10 |
|  | 100 | weak | 1 | H&N case #11 |
|  | 90 | weak | 1 | H&N case #12 |
|  | 100 | moderate | 2 | H&N case #13 |
|  | 90 | weak | 1 | H&N case #14 |
|  | 100 | weak | 1 | H&N case #15 |
|  | 80 | weak | 1 | H&N case #16 |
|  | 80 | weak | 1 | H&N case #17 |

Table: Area of in % of C/EBPα expressing cells and intensity scores of C/EBPα expression of

the positive area.
